# A Large Animal Model for Orthopedic Foot and Ankle Research

**DOI:** 10.3389/fvets.2022.816529

**Published:** 2022-02-03

**Authors:** Benjamin C. Gadomski, Kevin M. Labus, Holly L. Stewart, Katie T. Bisazza, Brad B. Nelson, Christian M. Puttlitz, Kirk C. McGilvray, Daniel P. Regan, Jeremiah T. Easley

**Affiliations:** ^1^Orthopedic Bioengineering Research Laboratory, Department of Mechanical Engineering and School of Biomedical Engineering, Colorado State University, Fort Collins, CO, United States; ^2^Preclinical Surgical Research Laboratory, Department of Clinical Sciences, Colorado State University, Fort Collins, CO, United States; ^3^Department of Microbiology, Immunology, and Pathology, Colorado State University, Fort Collins, CO, United States

**Keywords:** foot and ankle, sheep, carpus, fixation, syndesmosis

## Abstract

Trauma to the soft tissues of the ankle joint distal syndesmosis often leads to syndesmotic instability, resulting in undesired movement of the talus, abnormal pressure distributions, and ultimately arthritis if deterioration progresses without treatment. Historically, syndesmotic injuries have been repaired by placing a screw across the distal syndesmosis to provide rigid fixation to facilitate ligament repair. While rigid syndesmotic screw fixation immobilizes the ligamentous injury between the tibia and fibula to promote healing, the same screws inhibit normal physiologic movement and dorsiflexion. It has been shown that intact screw removal can be beneficial for long-term patient success; however, the exact timing remains an unanswered question that necessitates further investigation, perhaps using animal models. Because of the sparsity of relevant preclinical models, the purpose of this study was to develop a new, more translatable, large animal model that can be used for the investigation of clinical foot and ankle implants. Eight (8) skeletally mature sheep underwent stabilization of the left and right distal carpal bones following transection of the dorsal and interosseous ligaments while the remaining two animals served as un-instrumented controls. Four of the surgically stabilized animals were sacrificed 6 weeks after surgery while the remaining four animals were sacrificed 10 weeks after surgery. Ligamentous healing was evaluated using radiography, histology, histomorphometry, and histopathology. Overall, animals demonstrated a high tolerance to the surgical procedure with minimal complications. Animals sacrificed at 10 weeks post-surgery had a slight trend toward mildly decreased inflammation, decreased necrotic debris, and a slight increase in the healing of the transected ligaments. The overall degree of soft tissue fibrosis/fibrous expansion, including along the dorsal periosteal surfaces/joint capsule of the carpal bones was very similar between both timepoints and often exhibited signs of healing. The findings of this study indicate that the carpometacarpal joint may serve as a viable location for the investigation of human foot and ankle orthopedic devices. Future work may include the investigation of orthopedic foot and ankle medical devices, biologic treatments, and repair techniques in a large animal model capable of providing translational results for human treatment.

## Introduction

The foot is a highly complex portion of the human anatomy, consisting of 28 bones, 33 joints, and over 100 ligaments (1, 2). The foot articulates with long bones of the tibia and fibula via the ankle joint, with multiple tendons and muscles traversing the bones of the foot and ankle allowing for propulsion and balance. The ankle joint consists of the tibia, fibula and talus. The “distal syndesmosis” of the ankle joint is made up of the articulation of the distal tibia and fibula, held together by the anterior inferior tibiofibular ligament (AITFL), posterior inferior tibiofibular ligament (PITFL), interosseous ligament (IOL), and interosseous membrane (IOM). Trauma to these soft tissues often leads to syndesmotic instability, resulting in undesired movement of the talus, abnormal pressure distributions, and ultimately arthritis if deterioration progresses without treatment (3).

Amongst weight-bearing joints, ankle fractures are the most common with an estimated total of 673,214 ankle fractures occurring between 2012 and 2016 (3, 4). At the beginning of this period, the total estimated economic burden of foot and ankle procedures was ~$11 billion in the United States, up 38% from the previous decade (5). It has been reported that between 13 and 23% of all ankle fractures will have an associated syndesmosis injury (6–9). Typically, in ankle fractures, the AITFL, PITFL, IOL and a portion of the IOM are often torn in combination with the fracture. It also can occur as part of a “high ankle sprain” where one or more of the above ligaments are torn. Historically, syndesmotic injuries have been repaired by placing a screw across the distal syndesmosis to provide rigid fixation. While syndesmotic injuries are relatively common, questions remain about the most effective course of treatment, specifically if or when fixation screws should be removed (7). Trans-syndesmotic fixation with rigid metal screws has been accepted as the gold-standard of treatment. While rigid syndesmotic screw fixation immobilizes the ligamentous injury between the tibia and fibula to promote healing, the same screws inhibit normal physiologic movement and dorsiflexion (7), thus screw breakage is seen in many of these rigid constructs as a result of the physiologic motion that occurs across this joint once weight-bearing is restored (10, 11). Several studies have shown that intact screw removal can be beneficial for long-term patient success; however, the exact timing remains an unanswered question that necessitates further investigation, perhaps using animal models (7).

**Figure 1:**
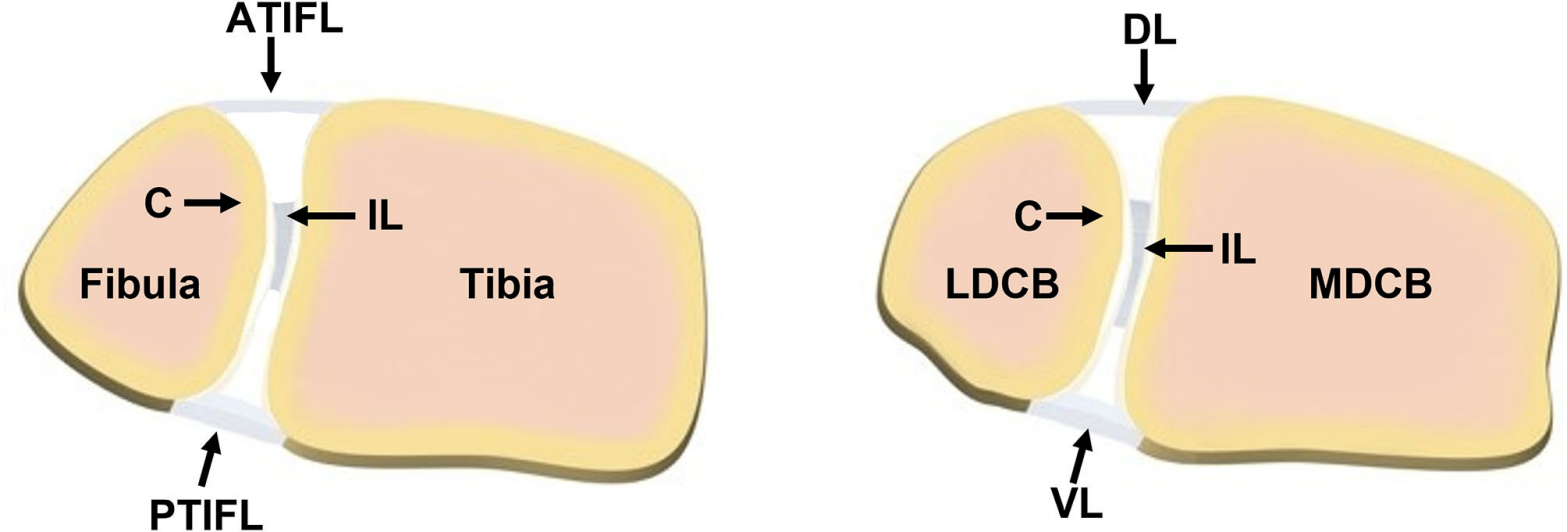
Comparison of the (left) human syndesmotic structure and the (right) sheep distal carpal bones. (Left) The human structure contains the fibula and tibia joined anteriorly by the anterior tibiofibular ligament (ATIFL), posteriorly by the posterior tibiofibular ligament (PTIFL), and centrally by an interosseous ligament (IL) located between a layer of cartilage (C) on the two bones. (Right) The distal carpal bones of the sheep (MDCB, medial distal carpal bone; LDCB, lateral distal carpal bone) share similar cross-sectional morphometry as the human fibula and tibia, ligamentous attachments (DL, dorsal ligament; VL, ventral ligament; IL, interosseous ligament) and cartilage (C) zone as the human syndesmotic complex. Illustration courtesy of Kelsea Erickson, DVM.

Use of animal models in foot and ankle research has been sparse. Though the findings related to basic science in small animal healing models for long bones and spine allow for insight into healing, they are not analogous to healing that may take place in different anatomic areas, such as the foot and ankle, due to differences in vascularity, soft tissue enclosure, and local biology (12). Lewis rats have been used to study fusion across the tibiotalar joint, which is believed to be the first dependable model of small animal ankle arthrodesis (12). C57BL/6J mice have been used as a model to study the effect of Metformin injection in the prevention of Achilles tendinopathy (13). Sprague-Dawley rats were used to study the injection of autologous adipose tissue-derived mesenchymal stem cells into their foot fat pad (14). While these animal studies have provided valuable insights, there remains a paucity of relevant animal models available for foot and ankle research. This is particularly true for large animal models, where appropriately sized implants and fixation devices may be investigated with more direct translation to human clinical practice. Sheep are commonly used in bone and joint research due to the similarities they share with humans as well as ease of handling and care (15–17). More specifically, in the context of syndesmosis, the front limb carpal bones of sheep share similar anatomy to humans because of the common anterior/dorsal, posterior/ventral, and interosseous ligamentous attachments between bones, similar morphometry of the medial/lateral distal carpal bones of the sheep to the human fibula/tibia, and a cartilage zone lining the respective bones in the interosseous region (Figure 1) (18). Due to these similarities and the need for relevant preclinical models, the purpose of this study was to develop a new, more translatable, large animal model that can be used for the investigation of clinical foot and ankle implants.

## Methods

### Surgical Procedure

**Figure 2:**
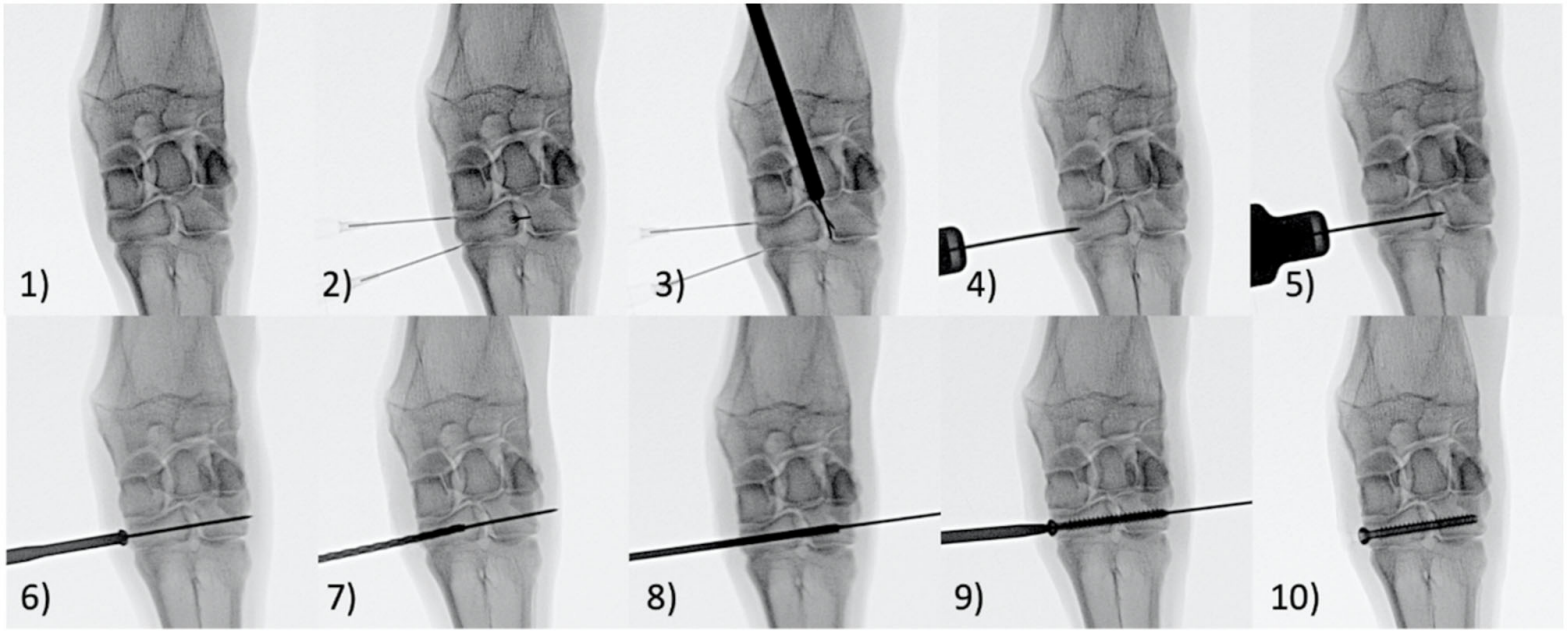
Step **(1)** An Anterior-posterior fluoroscopic image of the carpus was taken. Step **(2)** 22-gauge needles were placed within the carpometacarpal joint and the middle carpal joint as well as the intercarpal joint between the 2nd/3rd and 4th Carpal bones to aid in alignment. Step **(3)** A beaver blade was placed within the 2nd/3rd and 4th inter carpal bone to transect the anterior inter carpal ligament. Step **(4)** A 1.2mm K-wire was placed midway between the carpometacarpal joint and middle carpal joint. Step **(5)** The 1.2mm K-wire was inserted from the medial aspect of the fused 2nd/3rd carpal bone across the intercarpal joint and through the 4th carpal bone. The K-wire angled in ~15 degrees anteromedial to posterolateral direction in order to stay central within the 2nd/3rd and 4th carpal bones in both the dorsal and transverse planes. Step **(6)** A cannulated countersink tool was placed over the K-wire to countersink the medial edge of 2nd/3rd carpal bone. Step **(7)** The K-wire distance was measured and a 2.6mm cannulated drill bit was used to drill a hole of matching length. Step **(8)** A 4.0mm tap is placed over the K-wire to thread the drill hole. Step **(9)** A 4.0 mm cannulated screw was placed over the K-wire and driven across the fused 2nd/3rd and 4th carpal bones in a neutral position to stabilize the intercarpal joint. Step **(10)** The K-wire was removed from the cannulated screw.

This study was performed under the approval of Colorado State University's Animal Care and Use Committee (IACUC). Eight (8) skeletally mature female Rambouillet Cross sheep underwent stabilization of the left and right distal carpal bones (fused 2^nd^/3^rd^ and 4^th^) following transection of the dorsal intercarpal ligament (Figure 2) while the remaining two animals served as un-instrumented controls. Fentanyl patches (150 mcg) were placed transdermally on all animals 24 h prior to surgery. Additionally, all animals received florfenical (20 mg/kg, subcutaneous (SQ)) every 48 h for a total of 3 doses and Phenylbutazone (1 g, oral (PO)) every 24 h for a total of 7 doses starting 24 h prior to surgery. The auricular vein and artery were catheterized and anesthesia was induced with a combination of ketamine (3.3 mg/kg, intravenous (IV)) and diazepam (0.1 mg/kg, IV). Following anesthetic induction, the sheep were intubated with a cuffed endotracheal tube and maintained on isoflurane (1.5–3%) with 100% oxygen using positive pressure ventilation (20 cm H_2_O) for the duration of the procedure. Animals were placed under general anesthesia. Each animal was positioned in dorsal recumbency with the left front limb fully extended. The surgical site was clipped and aseptically prepared using chlorhexidine and isopropyl alcohol. Surgery was performed using sterile drapes and instruments and was performed aseptically. A single intraoperative dose of cefazolin (1 gram, IV) was administered within the first 15 min of the surgical procedure. Under fluoroscopic guidance via stab incisions only, the distal most row of carpal bones (2^nd^/3^rd^ carpal and 4^th^ carpal) were “stabilized” together using a cannulated screw. Prior to implanting the screws, the dorsal (anterior) ligaments were transected using a beaver blade by needle guidance determined with fluoroscopy, specifically, the dorsal ligament connecting the 2^nd^/3^rd^ carpal to the 4^th^ carpal bone. The two aforementioned bones in the distal row were connected by drilling a 1.2 mm K-wire into the medial aspect of the limb into the fused 2^nd^/3^rd^ carpal bones and extending into and through the 4^th^ carpal bone. A cannulated countersink was then created to accommodate the screw head. The k-wire distance was measured, and a 2.6 mm cannulated drill bit was used to drill a hole of matching length. The hole was then threaded using a 4.0 mm tap, and a 4.0 mm cannulated screw (Mini-Monster® Screw, Paragon 28®, Inc.) was driven across two distal carpal bones to stabilize the joint. The procedure was then repeated on the distal most row of carpal bones on the animal's contralateral limb. The stab incision was closed with 2–0 Nylon in a single horizontal mattress. A thick bandage with roll cotton, brown gauze and Elastkon was placed across both carpi to provide additional support and coverage to the carpus in the immediate recovery period. Following surgery and recovery, animals were allowed to roam freely and feed *ad libitum*. Four of the surgically stabilized animals were sacrificed 6 weeks after surgery (eight total 6-week Group specimens) while the remaining four animals were sacrificed 10 weeks after surgery (eight total 10-week Group specimens). The distal row of carpal bones were collected from the left and right limbs of two animals from an unrelated study and served as un-instrumented controls (four total Control group specimens). The distal row of carpal bones was carefully dissected from the left and right front limbs of each animal and high-resolution digital images and radiographs were taken of each specimen.

### Histological Preparation

The distal carpal bone specimen from each limb was sectioned into a block of tissue ~1 cm thick to display the bone-ligament-bone construct in the transverse plane using an irrigated band saw. This section region of interest (ROI) displayed the ligament, soft tissue, and surrounding bone. Samples were fixed in 10% neutral buffered formalin until fixation was complete. After fixation, tissue was decalcified in 8% trifluoroacetic acid and the decalcification end point was determined by daily radiographic assessment. Each sample was processed for decalcified histology using standard paraffin techniques and sectioned at 5 μm thickness. Slides were stained with Hematoxylin and Eosin (H&E). Two slides were cut through each joint ROI.

### Histomorphometry Analysis

**Figure 3:**
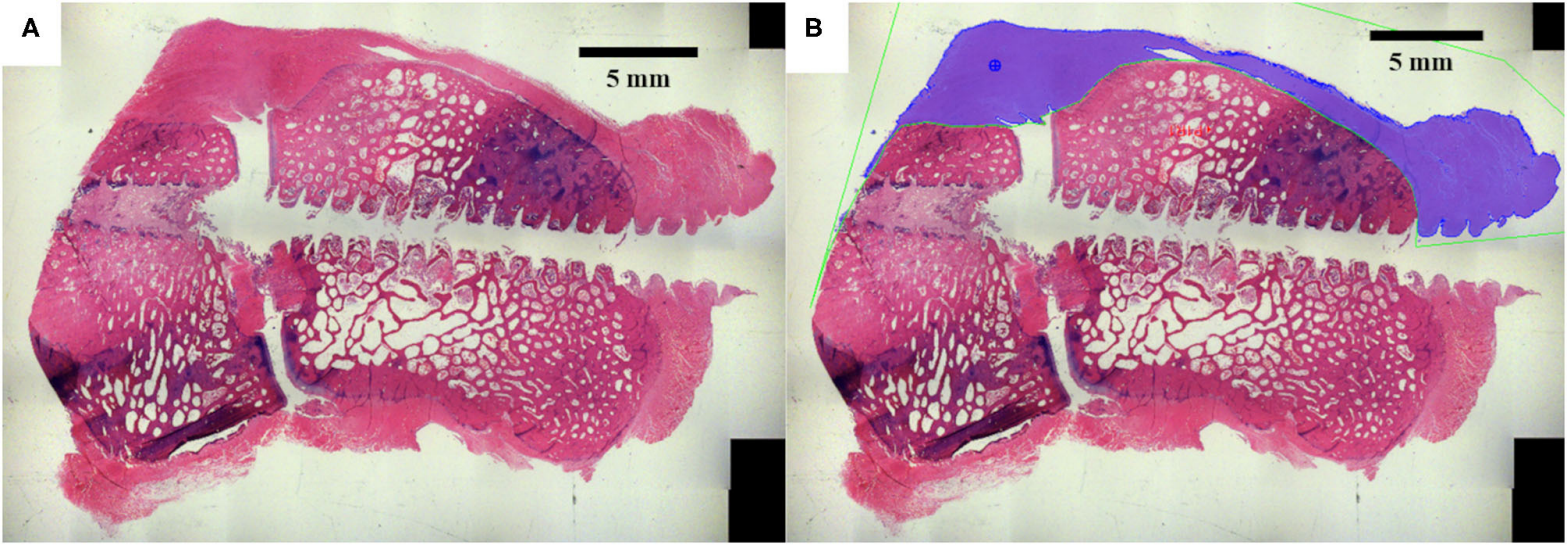
The dorsal ligament area was histomorphometrically analyzed. **(A)** Original histology section. **(B)** The dorsal ligament area was quantified around the dorsal peripheral surface of the carpal bones.

High-resolution digital images were acquired for all surgical site slides using a Nikon E800 microscope (AG Heinze, Lake Forest, CA), a Spot digital camera (Diagnostic Instruments, Sterling, Heights, MI), and ImagePro Premier Software (Media Cybernetics, Silver Spring, MD). The dorsal ligament area around the perimeter of the carpal bones was quantitatively assessed for all timetpoints (Figure 3) based on tissue stain color within the analyzed region (ImagePro Premier Software, Media Cybernetics, Silver Spring, MD). Statistical comparisons of histomorphometry data between treatment groups was performed using a one-way analysis of variance (ANOVA) with an alpha value of 0.05 and a Tukey *post-hoc* test (Minitab 17, Minitab Inc., State College PA).

### Histopathology

Histology slides were reviewed by a board-certified veterinary pathologist for analysis of cellular constituents and response using the ISO 10993-6 Annex E criteria for the biological evaluation of local effects following the implantation of a medical device. The outcome data included a quantitative scoring of the parameters listed in Table 1. The pathologist was initially blinded to the treatment parameters of each site and was unblinded following scoring of all the slides to draw conclusions between treatment groups.

**Table 1:** Semi-quantitative histopathology scoring parameters for the distal carpal bone histology slide sections. Scoring parameters are referenced from the ISO 10993-6 Annex E standards for the biological evaluation of local effects after implantation of medical devices.

	**Score**				
**Cell type/response**	**0**	**1**	**2**	**3**	**4**
Polymorphonuclear cells (PMNs)	None	Rare, 1–5/HPF*	5–10/HPF	Heavy infiltrate	Packed
Lymphocytes	None	Rare, 1–5/HPF	5–10/HPF	Heavy infiltrate	Packed
Plasma cells	None	Rare, 1–5/HPF	5–10/HPF	Heavy infiltrate	Packed
Macrophages (Mϕ)	None	Rare, 1–5/HPF	5–10/HPF	Heavy infiltrate	Packed
Giant cells	None	Rare, 1–2/HPF	3–5/HPF	Heavy infiltrate	Sheets
Necrosis	None	Minimal	Mild	Moderate	Severe
Neovascularization	None	Minimal	Mild	Moderate	Severe
Fibrosis	None	Minimal	Mild	Moderate	Severe
Collagen Fiber Organization	None	Very Loose	Moderate	Dense/Mature	N/A

*HPF – per high powered field.

## Results

### Surgical Procedure

All animals recovered well from surgery. Five of eight animals (63%) that underwent stabilization of the distal carpal bones showed mild to moderate, intermittent, shifting forelimb lameness that was managed with intermittent administration of Meloxicam (75 mg PO SID). Lameness was more prominent immediately after rising from lying down. No other postoperative complications were noted throughout the study and all sheep survived to their designated sacrifice timepoints. Procedural time for both carpi averaged 43 min from incision to closure (range 26–70 min). Surgery time decreased from an average of 53 min for the first 4 animals down to 31 min for the remaining animals. A total of 6/16 screws utilized measured 30 mm in length, 5/16 measured 32 mm in length and 5/16 measured 34 mm in length. Fluoroscopic guidance was highly valuable to the successful placement of the stabilizing screws.

### Radiographic Interpretation

Post-surgery radiographs revealed appropriate placement across the fixed 2^nd^/3^rd^ and 4^th^ carpal bones. Radiographs from explanted limbs and following disarticulation of the carpometacarpal joint revealed appropriate placement across both bones. All screws were placed from medial in a dorsolateral direction. This resulted in screws engaging more of the central portion of the fixed 2^nd^/3^rd^ carpal bones compared to the 4^th^ carpal bones. None of the screws bent or broke throughout the study period.

In 6-week radiographs, signs of severe arthritic changes (osteophyte formation, nonuniform joint space loss) were noted on dorsopalmar or lateromedial views in 1 of 8 (12.5%) carpi. This animal showed the most significant lameness of all animals on the study. 10-week radiographs noted 2 of 12 carpi (17% of carpi) with mild arthritic changes within the carpometacarpal joints. These two animals were also mildly lame on the arthritic limb.

### Histomorphometry

**Figure 4:**
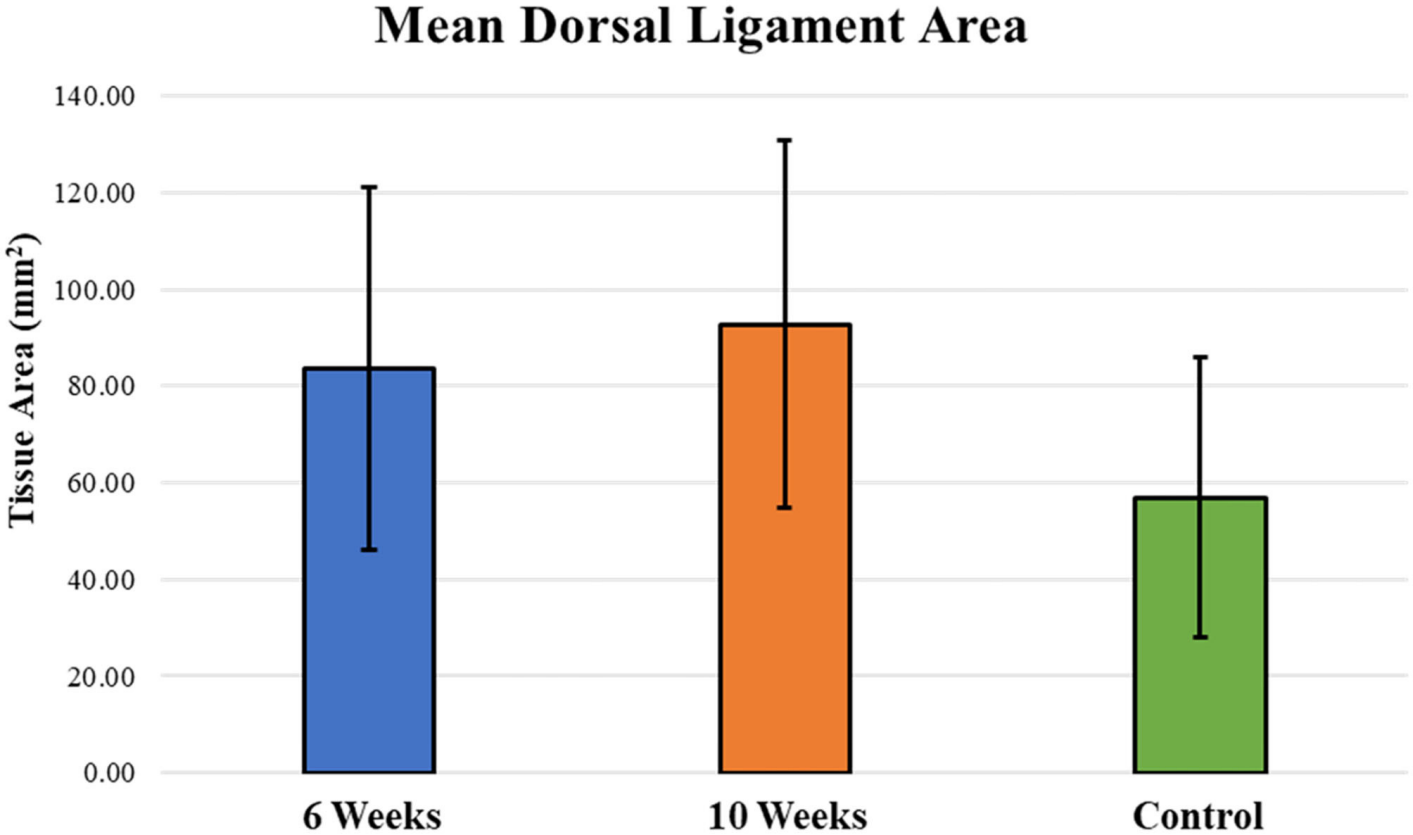
Mean dorsal ligament area around the periphery of the carpal bones was similar between 6-week and 10-week specimens and increased as compared to the Control group; however, those differences were not statistically significant between groups.

Mean dorsal ligament area around the periphery of the carpal bones was similar between 6-week and 10-week specimens (Figure 4). There was no statistically different change in mean dorsal ligament area in 6-week and 10-week groups as compared to the Control group. Bridging and reconnection of the severed dorsal ligament was observed in many of the specimens, with 62.5% (5 of 8 samples) demonstrating dorsal ligament bridging at 6 weeks and 87.5% (7 of 8 samples) demonstrating bridging at 10 weeks.

### Histopathology

**Figure 5:**
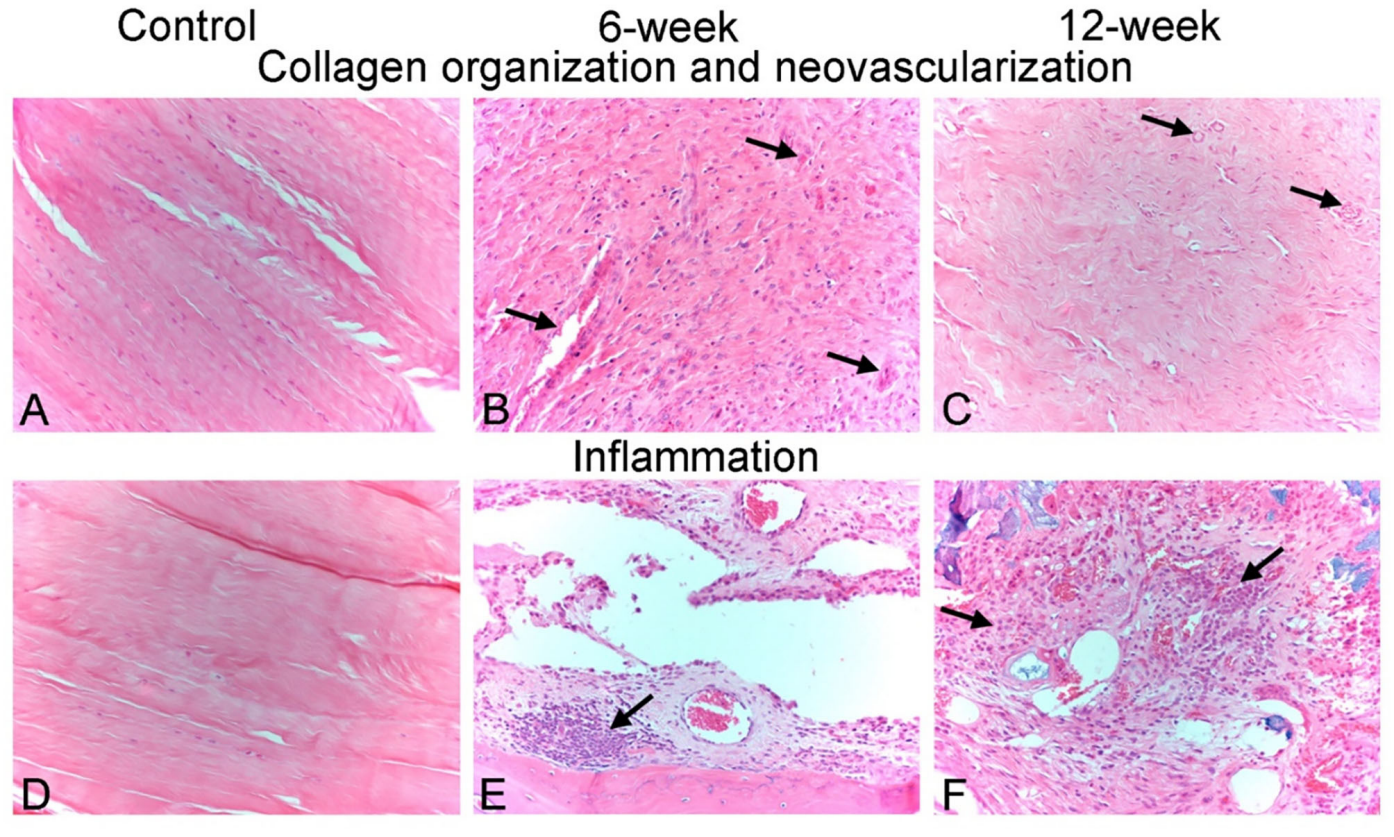
Representative photomicrographs of histological scoring parameters. **(A–C)** Collagen organization and neo-vascularization scoring. **(A)** Control animal 1 - left. Photomicrograph demonstrating a collagen fiber organization score of 3 (dense/mature) and a neovascularization score of 0 (none). Interosseous ligament collagen is comprised entirely of bundles of densely packed collagen fibers with a crimped appearance and which contain few flattened, hyperchromatic nuclei of ligament fibroblasts. No blood vessels are observed within this ligament tissue. **(B)** Animal PG04 - right, 6-week timepoint. Photomicrograph demonstrating a collagen fiber organization score of 0 (none/no organization) and a neovascularization score of 2 (mild). Newly produced fibrous tissue surrounding carpal bones is characterized by reactive and disorganized fibroplasia with haphazardly intersecting collagen bundles, and numerous plump reactive fibroblasts. 3–5 capillary-sized neo-vessels per high powered (400x magnification) field are present throughout this fibrous tissue (arrows). **(C)** Animal PG07 - right, 12-week timepoint. Photomicrograph demonstrating a collagen fiber organization score of 2 (moderate) and a neovascularization score of 2 (mild). Newly produced fibrous tissue is being progressively re-organized into more parallel and tightly packed bundles of collagen fibers, similar to native interosseous ligament observed in healthy control animals. Similar to the 6-week animal shown in **(B)**, 3–5 capillary-sized neo-vessels per high powered (400x magnification) field remain present throughout this fibrous tissue (arrows). **(D**–**F)** Inflammation scoring. **(D)** Control animal 4 - left. Photomicrograph demonstrating a cumulative inflammation score 0. No inflammatory cells were observed in the carpal ligament tissues of any healthy control animals. **(E)** Animal PG06 – right, 6-week timepoint. Photomicrograph demonstrating a cumulative inflammation score of 6/20. Multifocal nodular clusters of predominately lymphocytes and fewer plasma cells (arrow), as well as scattered individual infiltrating macrophages, were observed throughout the newly produced fibrous tissue. **(F)** Animal PG05 – left, 12-week timepoint. Photomicrograph demonstrating a cumulative inflammation score of 8/20. Similar to animal PG06-right, clusters of infiltrating lymphocytes, plasma cells and macrophages (arrows) are multifocally present throughout the newly produced fibrous tissue. All images are 20x magnification.

All evaluated histopathological parameters were predominately similar in composition and severity across all study animals and timepoints (Table 2). Representative histologic images of each timepoint are presented in Figure 5. Overall, animals sacrificed at 10 weeks post-surgery had a slight trend toward mildly decreased inflammation, decreased necrotic debris, and a slight increase in the healing and organization of the reactive fibrous tissue surrounding the interosseous ligament. Mean (±standard deviation) cumulative inflammation scores (the sum of polymorphonuclear cells, lymphocytes, plasma cells, macrophages, and giants cells) were 5(±3) and 4(±2) for the 6 and 10-week groups, respectively, with 0 being the lowest and 20 being the highest possible scores. The carpal joint of the 6-week group that displayed severe arthritic changes had a cumulative inflammation score of 12. The two animals that showed mild arthritic changes at 10 weeks had cumulative inflammation scores of 3 and 8. Several carpal joints from both timepoints showed cumulative inflammation scores as great as 6 with no apparent signs of arthritic progression. The overall degree of soft tissue fibrosis/fibrous expansion, including along the dorsal periosteal surfaces/joint capsule of the carpal bones was very similar between both timepoints.

**Table 2:** Mean and (standard deviation) histopathologic scores for Control, 6-week, 10-week specimens. PMN indicates polymorphonuclear cells, lymphs indicates lymphocytes, Mj indicates macrophage cell presence, neovasc indicates neovascularization.

**Timepoint**	**PMNs**	**Lymphs**	**Plasma cells**	**Mj**	**Giant cells**	**Cumulative inflammation score**	**Osteoblast activity**	**Osteoclast Activity**	**Necrosis**	**Neovasc**	**Fibrosis**	**Collagen fiber organization**
Control	0 (0)	0 (0)	0 (0)	0 (0)	0 (0)	0 (0)	0 (0)	0 (0)	0 (0)	0 (0)	0 (0)	3 (0)
6 Weeks	1 (1)	1 (1)	1 (1)	1 (0)	0 (0)	5 (3)	1 (1)	1 (1)	2 (1)	2 (1)	2 (1)	1 (1)
10 Weeks	0 (0)	1 (0)	1 (1)	1 (1)	1 (1)	4 (2)	2 (1)	1 (1)	1 (1)	2 (1)	2 (1)	2 (1)

The primary histological changes within the tissues of animals from both timepoints were most evident in the void space left at the site of screw placement. This void space bisected both carpal bones, traversed articular surfaces, and spanned the intraarticular space. While not factored into the overall histological score for each section, the host tissue response within this screw void space almost always communicated with either or both the interosseous ligament present between the carpal bones, as well as the fibrous periosteum/joint capsule/ligaments along the dorsal (cranial) periosteal surface of the carpal bones. Thus, the nature of the tissue response within this screw placement space often extended to characterize that present within the tissue regions-of-interest (ROI) of the intra-articular space, interosseous ligament, and soft tissues along the dorsal aspect of the carpal bones.

Regarding the interosseous ligament and the associated soft tissue response surrounding it and filling the intra-articular space, this response was again typically an extension of the fibrous tissue which surrounded the site of the screw placement. In the majority of animals and sites, the interosseous ligament was intact and primarily retained its native structure, with the majority of the ligamentous structure appearing histologically normal, and the only host tissue response occurring along the peripheral/lateral margins of the ligament.

## Discussion

In many situations, *in vitro* and cadaveric investigations may be sufficient to answer the relevant research questions of the particular study, however, limitations exist in these methods, and live animal research becomes necessary to elucidate translational, physiologically accurate responses. In translational orthopedic research, these physiologic responses may include inflammatory reactions as a result of device or drug safety, bone remodeling, cartilage generation/degeneration, and the overall healing response in a complex, multivariable systemic environment. Furthermore, live animal models that are clinically and anatomically relevant, especially in larger species, are vital to the regulatory approval and commercialization process of orthopedic medical devices. To date, relevant animal models for orthopedic foot and ankle research have been scarce. Small animal models, specifically mice and rats, have been the historical gold standard in this field. While insights gained using these animal models have been valuable, these small animal foot and ankle models possess shortcomings that prevent meaningful translation to the human clinical setting including vastly different metabolic rates, healing rates, bone structures, and perhaps most importantly, extremely different physical sizes from humans that prevent the direct investigation of implants designed for human use (19). This study sought to develop a new, large animal model for foot and ankle research leveraging the orthopedic resemblances between sheep and humans. Sheep are commonly used in bone and joint research due to the similarities they share with humans, specifically similar weight, metabolic rates, healing rates, bone microarchitecture, and anatomical size, as well as ease of handling and care (15–17, 19). Importantly, the anatomical size of sheep allows for the direct investigation of orthopedic devices that are sized specifically for human clinical use. This study presents the first large animal (sheep) model for foot and ankle research using orthopedic hardware sized for human use in the distal carpal bones of the sheep forelimb.

There is limited description of sheep carpal anatomy specifically in the literature (20). However, the carpal anatomy of sheep is similar to that of the cow. There are three joints within the sheep carpus, consisting of the: (1) antebrachial joint between the radius/ulna and proximal carpal bones, (2) middle carpal joint between the two rows of carpal bones and (3) the carpometacarpal joint between the distal carpal bones and the proximal metacarpals. The proximal row of carpal bones consists of the medially located radial carpal bone, the centrally located intermediate carpal bone and the laterally located ulnar and accessory carpal bones. The distal row of carpal bones consists of a medially located fused 2^nd^ and 3^rd^ carpal bones and a laterally located 4^th^ carpal bone. The 1^st^ carpal bone and 5^th^ carpal bone are not present. While the carpal anatomy of the sheep does not mimic the carpal or tarsal anatomy of humans, carpal and tarsal joints of both species consist of cuboid bones or squared shaped bones. These cuboid bones are commonly stabilized or fused with internal screw fixation in people in a similar fashion to the procedure described herein. In this current study, the carpal bones in the sheep model were selected because they mimic the general structure and axial loading pattern of the syndesmosis. Specifically, ligaments are present anterior and posterior to the joint, as well as having an interosseous ligament. The sizes of the bones are similar to that of the short bones of the foot, allowing for implants manufactured for foot and ankle procedures to be used in this region.

The animals of this study demonstrated high tolerance to the surgical procedure with only mild, intermittent lameness in the operated limbs. The orthopedic hardware used in this study, a 4.0 mm cannulated screw, was a commercially available device used in human foot and ankle procedures. The screw was sized appropriately for the anatomy of the sheep carpometacarpal bones, and hardware failure was not experienced at the surgical site. The ability to directly investigate the effects of orthopeadic devices designed specifically for human use is a distinct advantage of this new sheep model.

In terms of the overall healing response, similarities were observed between the animals in this study and what has been documented in human patients that underwent trans-syndesmotic fixation. First, more than half of the animals in this study demonstrated bridging and reconnection of the transected dorsal ligament at the 6-week timepoint, and all but one specimen demonstrated reconnection of the dorsal ligament at the 10-week timepoint. The soft tissues surrounding the dorsal perimeter of the carpal bones in the animals of this study generally displayed well-organized collagen structures despite the inflammatory response induced by the surgical procedure and ligament transection. This organization tended to decrease toward the intra-articular space between the carpal bones. This healing ability has also been shown to be characteristic of the anterior tibiofibular ligament in human patients with acute syndesmotic injuries as trans-fixation screws are often removed post-operatively following a recovery period without concomitant widening of the syndesmosis (21). Arthritic changes were observed in 1 of 8 (12.5%) carpi at the shorter timepoint (6 weeks) and 2 of 12 carpi (17%) at the 10-week timepoint. This also is not unlike the human clinical presentation of syndesmotic instability and fixation, and trans-syndesmotic fixation has been shown to be a risk factor associated with human synostosis (22). Further, while related but not directly investigated in this animal study, it is well known that malreduction of the syndesmosis can play a major role in ankle joint arthritis in humans (23). Overall inflammation scores decreased in the areas around the stabilization screw and dorsal ligaments over time from 6 to 10 weeks in the animals of this study, and while it is impossible to directly compare these results to histopathological inflammatory response of human patients, it is likely that arthritic changes are also associated with some level of inflammatory response.

There are, of course, inherent limitations when using animals as a surrogate for clinical human research. These shortcomings include postural (quadruped vs. biped) and healing differences. Perhaps most importantly, the constraints of human clinical research prevent the direct histological comparison of the healing response of the ligaments in the human syndesmosis to the histopathological observations from this study. Despite these limitations, numerous similarities between sheep and humans, including anatomical characteristics (similar bone sizes), *in vivo* biomechanical loads, bone composition, bone macro and micro architecture, and ligamentous healing characteristics, make the sheep model one of the most translatable for orthopedic research.

In conclusion, the findings of this study indicate that the carpometacarpal joint may serve as a viable location for the investigation of human foot and ankle orthopedic devices. Future work may include the investigation of orthopedic foot and ankle medical devices, biologic treatments, and repair techniques in a large animal model capable of providing translational results for human treatment.

## Data Availability Statement

The raw data supporting the conclusions of this article will be made available by the authors, without undue reservation.

## Ethics Statement

The animal study was reviewed and approved by Colorado State University Institutional Animal Care and Use Committee.

## Author Contributions

BG participated in conceptual design of the study, data acquisition, analysis, interpretation, manuscript drafting, and final approval. KL participated in data acquisition, analysis, interpretation, manuscript drafting, and final approval. HS, KB, and BN participated in data acquisition, manuscript drafting, and final approval. CP participated in conceptual design of the study, manuscript drafting, and final approval. KM participated in conceptual design of the study, manuscript drafting, and final approval. DR participated in data acquisition, analysis, interpretation, manuscript drafting, and final approval. JE participated in conceptual design of the study, data acquisition, analysis, interpretation, manuscript drafting, and final approval. All authors agree to be accountable for this work and ensure its integrity.

## Funding

This work was funded by a research grant from Paragon 28, LLC., Englewood, CO.

## Conflict of Interest

The authors declare that the research was conducted in the absence of any commercial or financial relationships that could be construed as a potential conflict of interest.

## Publisher's Note

All claims expressed in this article are solely those of the authors and do not necessarily represent those of their affiliated organizations, or those of the publisher, the editors and the reviewers. Any product that may be evaluated in this article, or claim that may be made by its manufacturer, is not guaranteed or endorsed by the publisher.
